# Why Epidemics Rage at Night

**Published:** 1850-09

**Authors:** 


*Why Epidemics Rage at Night.*—It was in one night that 4,000 perished in the plague of London of 1605. It was at night that the army of Sennacherib was destroyed. Both in England and on the continent a large portion of the cholera cases, in its several forms, have been observed to have occurred between one and two o'clock in the morning. The "danger of exposure to night air" has been a theme of physicians from time immemorial; but it is remarkable that they have never called in the aid of chemistry to account for the fact.

It is at night that the stratum of the air nearest the ground must always be the most charged with the particles of animalized matter given out from the skin, and deleterious gases such as carbonic acid gas, the product of respiration, and sulphuretted hydrogen, the product of the sewers. In the day, gases and vaporous substances of all kinds rise in the air by the rarefaction of heat; at night, when this rarefaction leaves them, they fall by an increase of gravity, if imperfectly mixed with the atmosphere, while the gases evolved during the night, instead of ascending, remain at nearly the same level. It is known that carbonic acid gas, at a low temperature, partakes so nearly of the nature of a fluid, that it may be poured out of one vessel into another; it rises at the temperature at which it is exhaled from the lungs, but its tendency is towards the floor or the bed of the sleeper, in cold and unventilated rooms.—*New York Medical Gazette.*

